# Financial Resources and Biomarkers of Aging in the National Health and Aging Trends Study

**DOI:** 10.1093/geroni/igaa057.1974

**Published:** 2020-12-16

**Authors:** Laura Samuel, Laken Roberts, Danielle Boyce, Melissa Hladek, Sarah LaFave, Sarah Szanton

**Affiliations:** 1 Johns Hopkins University, Baltimore, Maryland, United States; 2 Johns Hopkins University School of Nursing, Baltimore, Maryland, United States

## Abstract

Lower income and financial strain (i.e. difficulty making ends meet) are associated with worse aging biomarkers, but evidence among nationally representative samples is limited. This cross-sectional study tested whether income to poverty ratio (analyzed separately for those <500% vs. ≥500% poverty threshold) and financial strain are associated with biomarkers of aging among NHATS participants aged ≥65 years (n=4,648), adjusting for age, race/ethnicity, gender, smoking, BMI, and diabetes diagnosis for hemoglobin A1c. Sampling weights were applied. Among those with incomes <500% poverty, higher income was associated with lower hemoglobin A1c (b= -0.0196, p=0.007), CMV (b= -0.0689, p<0.001) and CRP (b= -0.0428, p=0.012). Among those with incomes ≥500%, higher income was associated with lower IL-6 (b= -0.0001, p=0.023) and lower CMV (b= -0.0001, p<0.001). Financial strain was not associated with biomarkers. Income is more strongly associated with biomarkers among the lower income group, calling for special attention to this vulnerable population.

